# Genomic signatures of *Mannheimia haemolytica* that associate with the lungs of cattle with respiratory disease, an integrative conjugative element, and antibiotic resistance genes

**DOI:** 10.1186/s12864-016-3316-8

**Published:** 2016-11-29

**Authors:** Michael L. Clawson, Robert W. Murray, Michael T. Sweeney, Michael D. Apley, Keith D. DeDonder, Sarah F. Capik, Robert L. Larson, Brian V. Lubbers, Brad J. White, Theodore S. Kalbfleisch, Gennie Schuller, Aaron M. Dickey, Gregory P. Harhay, Michael P. Heaton, Carol G. Chitko-McKown, Dayna M. Brichta-Harhay, James L. Bono, Timothy P. L. Smith

**Affiliations:** 1 United States Department of Agriculture, Agricultural Research Service, U.S. Meat Animal Research Center, Clay Center, NE USA; 2 Zoetis, Kalamazoo, MI USA; 3 Kansas State University, Manhattan, KS USA; 4 Veterinary and Biomedical Research Center, Inc, Manhattan, KS USA; 5 University of Louisville, Louisville, KY USA

**Keywords:** *Mannheimia*, *haemolytica*, Genomics, Polymorphisms, Genotypes, Subtypes, Antibiotics, Bovine, Respiratory, Disease

## Abstract

**Background:**

*Mannheimia haemolytica* typically resides in cattle as a commensal member of the upper respiratory tract microbiome. However, some strains can invade their lungs and cause respiratory disease and death, including those with multi-drug resistance. A nucleotide polymorphism typing system was developed for *M. haemolytica* from the genome sequences of 1133 North American isolates, and used to identify genetic differences between isolates from the lungs and upper respiratory tract of cattle with and without clinical signs of respiratory disease.

**Results:**

A total of 26,081 nucleotide polymorphisms were characterized after quality control filtering of 48,403 putative polymorphisms. Phylogenetic analyses of nucleotide polymorphism genotypes split *M. haemolytica* into two major genotypes (1 and 2) that each were further divided into multiple subtypes. Multiple polymorphisms were identified with alleles that tagged genotypes 1 or 2, and their respective subtypes. Only genotype 2 *M. haemolytica* associated with the lungs of diseased cattle and the sequence of a particular integrative and conjugative element (ICE). Additionally, isolates belonging to one subtype of genotype 2 (2b), had the majority of antibiotic resistance genes detected in this study, which were assorted into seven combinations that ranged from 1 to 12 resistance genes.

**Conclusions:**

Typing of diverse *M. haemolytica* by nucleotide polymorphism genotypes successfully identified associations with diseased cattle lungs, ICE sequence, and antibiotic resistance genes. Management of cattle by their carriage of *M. haemolytica* could be an effective intervention strategy to reduce the prevalence of respiratory disease and supplemental needs for antibiotic treatments in North American herds.

**Electronic supplementary material:**

The online version of this article (doi:10.1186/s12864-016-3316-8) contains supplementary material, which is available to authorized users.

## Background

Bovine respiratory disease (BRD) is a worldwide animal health and welfare problem that costs the United States cattle industry over one billion dollars annually [1–4]. Multiple viral and bacterial pathogens, as well as host and environmental factors either cause or contribute to BRD (reviewed in [5]). *M. haemolytica* is a predominant bacterial agent of BRD that causes severe fibrinonecrotic pneumonia in both beef and dairy cattle [3, 6] and historically has been the bacteria species most commonly isolated from the lungs of cattle afflicted with respiratory disease [5, 7–9].

*M. haemolytica* is an opportunistic pathogen that often resides in the upper respiratory tract of cattle as a commensal, and invades the lower respiratory tract when animals are immunocompromised by viral infections, stress, or other factors (reviewed in [10]). Typing of *M. haemolytica* strains based on capsular phenotypes and pulsed-field gel electrophoresis (PFGE) have both indicated that *M. haemolytica* is comprised of distinct sub-species, or strain types, that do not all equally associate with BRD [10]. Strains with either A2 or A4 capsular serotypes have been more frequently isolated from the upper respiratory tracts of cattle without signs of respiratory disease, while strains with A1 or A6 serotypes have been more frequently isolated from the lungs and nasopharynx of diseased animals [3, 10–12]. Similarly, a dendrogram of PFGE profiles from *M. haemolytica* strains isolated from the nasopharynx of cattle with or without signs of respiratory disease was found to have two major clusters that disproportionately represented isolates from animals with and without signs of BRD, respectively [13].

*M. haemolytica* strains also demonstrate diverse susceptibility phenotypes to antimicrobials used to treat BRD cases. Overall, strains isolated from North American cattle with respiratory disease over the last several decades have trended towards decreasing susceptibilities to some of the antimicrobials used for treatment [14–17]. Conversely, *M. haemolytica* isolated from the upper respiratory tract of cattle without clinical signs of respiratory disease have tended to display lower overall antibiotic resistance than strains from diseased animals [16].

*M. haemolytica* and other members of the *Pasteurellaceae* family that cause BRD can harbor plasmids or ICEs containing antibiotic resistance encoding genes [18–25]. Plasmids containing antibiotic-resistance genes have been recognized in *M. haemolytica* for over 30 years [26]. In contrast, ICEs have been recognized in BRD-causing pathogens only recently. An ICE was first identified in *Pasteurella multocida* in 2012 (ICE*Pmu1*) that was isolated from a beef calf in Nebraska and contained two regions encoding a total of 11 antibiotic resistance genes [22]. Under laboratory conditions, ICE*Pmu1* was able to transfer from *P. multocida* to *M. haemolytica* by conjugation and integration [22]. Homologs of ICE*Pmu1,* such as ICE*Mh1*, have subsequently been identified in field-collected isolates of *M. haemolytica* from cattle in Nebraska, Pennsylvania, and Texas [21, 23, 27, 28]. The homologs share a conserved backbone that consists of blocks of genes which are adjacent to antibiotic resistance gene regions [23]. The number and assortment of antibiotic resistance genes vary between homologs, and not all contain resistance genes. For example, ICE*Mh1* has five antibiotic resistance genes, while yet another putative homolog found in *M. haemolytica* strain USDA_ARS_USMARC 183 has none [23]. Homologs to ICE*Pmu1* and ICE*Mh1* that contain antibiotic resistance genes do not appear restricted to one geographical region of North America [21, 27, 29], and may have a wide distribution and role in *M. haemolytica* antibiotic resistance. Given that capsular serotypes and PFGE profiles both indicate that *M. haemolytica* strains do not all equally associate with BRD, it is plausible that homologs containing antibiotic resistance genes could be more prevalent in *M. haemolytica* strains that associate with disease, as they would be more frequently exposed to BRD prevention and control measures that include antimicrobials.

The aim of this study was to investigate *M. haemolytica* at the subspecies level for associations with diseased cattle lungs, the conserved backbone of ICE*Pmu1,* ICE*Mh1,* and other ICE homologs, and antibiotic resistance genes found within variable regions of ICE*Pmu1* and ICE*Mh1.* Accordingly, there were three major goals. The first was to sequence the genomes of 857 *Mannheimia haemolytica* isolates, including isolates from the lungs of diseased cattle that were located throughout much of North America, and isolates from the nasopharynx of cattle without clinical signs of respiratory disease. The second was to identify nucleotide polymorphisms that type *M. haemolytica* of North American cattle into well-supported clades using the sequences generated in this study, and those available from an additional 276 isolates. The third was to test nucleotide polymorphism-derived clades for associations with the lungs of cattle with respiratory disease, the conserved backbone of ICE*Pmu1,* ICE*Mh1*, and other related homologs, and antibiotic resistance genes harbored on ICE*Pmu1* and ICE*Mh1*.

## Results

### Isolate sequencing coverage and classification into groups

A total of 857 *M. haemolytica* isolates from North American cattle were sequenced to a minimum level of 10× sequence coverage per genome. Another 276 isolates from North American cattle that were previously sequenced at the same level of coverage were also included in this study [29, 30]. The isolates were arranged into two collections, Zoetis and KSU-USMARC, that each consisted of two groups (Table 1, Additional file 1). The two groups of the Zoetis collection (lung clinical isolates 1 and 2) were each comprised of epidemiologically-unrelated isolates from the lungs of cattle with BRD, however, isolates between the groups shared some epidemiological relatedness. Within the KSU-USMARC collection, one group (nasopharyngeal non-clinical isolates) consisted of non-clinical isolates from the nasopharynx of cattle without BRD signs that were selected for minimal epidemiological relatedness. The other group (clinical and non-clinical isolates) consisted of isolates from the lungs or nasopharynx of cattle with or without signs of respiratory disease. Many isolates within the clinical and nonclinical group were epidemiologically related to each other.

**Table 1:** Isolates used for whole genome sequencing and/or analyses

Collection	Group	# Isolates	States/provinces	Year/range	Statistical test use
Zoetis	Lung Clinical Isolates 1	155	35/5	2002–2011	Yes^a^
Zoetis	Lung Clinical Isolates 2	162	29/3	2002–2011	No^b^
KSU-USMARC	Nasopharyngeal non-Clinical Isolates	35	3/0	2013	Yes^c^
KSU-USMARC	Clinical and non-Clinical Isolates^d^	781	4/0	2013	No^e^

^a^Used for association testing of genotype 2 *M. haemolytica* with the lungs of cattle with respiratory disease and ICE sequence, and association testing of 2b *M. haemolytica* with antibiotic resistance genes encoded by ICE*Pmu1* and/or ICE*Mh1*

^b^Not used for statistical tests due to epidemiological overlap with lung clinical isolates group 1

^c^Used for association testing of genotype 2 *M. haemolytica* with the lungs of cattle with respiratory disease and ICE sequence

^d^Represented by both lung and nasopharyngeal isolates. Previously sequenced isolates are included in this group (*n* = 276), [29, 30]. All other isolates of both collections were sequenced in this study (*n* = 857)

^e^Not used for statistical tests due to epidemiological overlap among isolates within the group and with the group of nasopharyngeal non-clinical isolates

### Identification of nucleotide polymorphisms and *M. haemolytica* genotypes 1 and 2

**Fig. 1:**
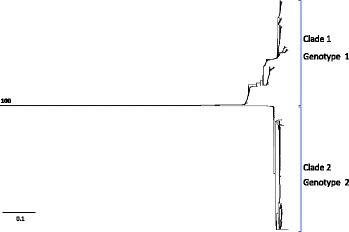
Neighbor-Joining tree of concatenated nucleotide-polymorphism genotypes from 1133 *M. haemolytica* isolates. The scale bar represents substitutions per site. The number within the tree represents bootstrap level support for the separation of clades one and two

Prior to quality control filtering, 48,403 putative polymorphisms were identified from the sequences of all 1133 isolates included in the study. Of those, 26,081 polymorphisms were selected for further analyses that were not: 1) in duplicated genome or integrated phage regions of reference *M. haemolytica* strain USDA-ARS-USMARC-183, 2) singletons (single observation of alternative allele), or 3) ambiguously genotyped for more than 5% of the isolates. To generate a Neighbor-Joining tree that would be minimally influenced by missing data, the 26,081 polymorphisms were additionally screened for those with allele genotype call rates of 95% or higher for the 1133 isolates, which yielded 16,447 polymorphisms (Additional files 2 and 3). The tree of concatenated polymorphism genotypes depicted two deeply divided clades with strong bootstrap support (Fig. 1). Of the 16,447 polymorphisms used to generate the tree, 13,941 of them separated the two clades, where each allele was found exclusively in one of the clades. These two clades represent two major genotypes of *M. haemolytica.*

### Identification of subtypes and tagging nucleotide polymorphisms within genotype 1

**Fig. 2:**
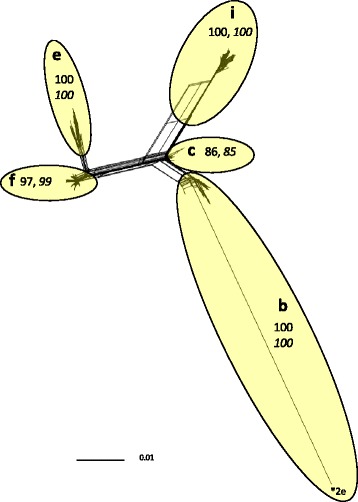
Neighbor-Joining network of concatenated nucleotide-polymorphism genotypes from 521 *M. haemolytica* genotype 1 isolates. Letters and ellipsoids within the network represent subtypes and their boundaries, respectively. Non-italicized numbers within the network represent maximum-likelihood support, and italicized numbers represent bootstrap support. The asterisk represents recombinant sequence of 2e on the backbone of 1b. The scale bar represents substitutions per site

The original set of 26,081 polymorphisms was re-evaluated within 521 of 525 genotype 1 isolates classified in this study to identify polymorphisms suitable for higher resolution typing of genetic diversity within the genotype. Four genotype 1 isolates were excluded from the analysis due to missing data. Of the original 26,081 polymorphisms, 16,888 had a 90% or higher allele call rate for the 521 isolates (Additional files 4 and 5). A total of 1710 of the 16,888 polymorphisms were informative for the identification of subtypes within genotype 1, meaning that both polymorphism alleles were observed in genotype 1 isolates (Additional file 6). A phi test for recombination applied to concatenated polymorphism alleles of the 521 isolates was positive (*p* = 0.0). Accordingly, a Neighbor-Joining network, which accounted for recombination [31], was constructed from concatenated polymorphism alleles of each isolate (Fig. 2). Five clusters were observed that were all well-supported by Shimodaira-Hasegawa approximate likelihood ratio test (SH-aLRT) values that exceeded 80%, and with four of the clusters additionally supported by ultrafast bootstrap values exceeding 95%. Of all 525 genotype 1 isolates classified in this study, 515 (98%) placed unambiguously within one of the five clusters. Only ten isolates were not confidently placed due to missing data and/or inadequate statistical support within a cluster (Additional file 1). The five clusters represent 5 genetic divisions, or subtypes of genotype 1 *M. haemolytica* (1b, 1c, 1e, 1f, 1i), (Fig. 2).

Tagging nucleotide polymorphisms were identified for all five subtypes of genotype 1. These were polymorphisms where one allele was exclusively observed in all isolates of a particular subtype: (1b; *n* = 153), (1c; *n* = 2), (1e; *n* = 106), (1f; *n* = 5), (1i; *n* = 349), (Additional file 6). Many of the tagging polymorphism were identified in close proximity to each other, indicating probable sites of recombination [32].

### Identification of subtypes and tagging polymorphisms within genotype 2

**Fig. 3:**
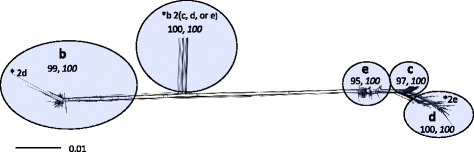
Neighbor-Joining network of concatenated nucleotide-polymorphism genotypes from 600 *M. haemolytica* genotype 2 isolates. Letters and ellipsoids within the network represent subtypes and their boundaries, respectively. Non-italicized numbers within the network represent maximum-likelihood support, and italicized numbers represent bootstrap support. The scale bar represents substitutions per site. Asterisks indicate recombination: (2b with 2d), (2b with 2c, 2d, or 2e), (2d with 2e)

The original set of 26,081 polymorphisms was also re-evaluated within 600 of 608 genotype 2 isolates classified in this study to identify polymorphisms suitable for higher resolution typing of genetic diversity within genotype 2. Eight genotype 2 isolates were excluded from the analysis due to missing data. From the initial 26,081 polymorphisms, 24,475 had alleles observed at a frequency of 90% or higher for the 600 genotype 2 isolates. (Additional file 5). A total of 3499 of the polymorphisms were informative for genotype 2 (Additional files 7 and 8). As in genotype 1, the concatenated polymorphism alleles of genotype 2 isolates tested strongly positive for recombination (phi test *p* = 0.0). A Neighbor-Joining network constructed from the concatenated polymorphism allele genotypes of each of the 600 isolates yielded 5 clusters that were all well supported by both SH-aLRT and ultrafast boot strap values (Fig. 3). Four clusters were assigned as subtype representatives (b, c, d, e). The one cluster not assigned to a subtype was primarily a 2b subtype that had recombined with either a 2c, 2d, or 2e subtype (designated *b 2 (c, d, or e) in Fig. 3), and was only represented by the concatenated nucleotide polymorphism genotypes of three isolates (Additional file 1). Recombination was also detected between subtypes 2b and 2d, as well as subtypes 2d and 2e (Fig. 3).

A total of 598 of all 608 genotype 2 isolates classified in this study (98%) unambiguously placed in subtypes b, c, d, or e, or were identified as recombinants between the four subtypes. Ten genotype 2 isolates were not assigned subtypes due to missing data and/or lack of statistical support within a cluster. Tagging polymorphisms were identified for subtype 2b (*n* = 1574), 2c (*n* = 7), and 2d (*n* = 158), (Additional file 9). None were found for subtype 2e, which could represent an ancestral state to the other subtypes.

### Association of genotype 2 *M. haemolytica* with the lungs of cattle with respiratory disease

**Fig. 4:**
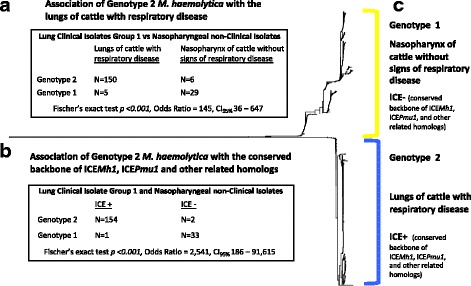
*M. haemolytica* genotype 2 associations. Section (**a**) shows a 2-by-2 table of 190 isolates categorized by their genotypes and origin from either the lungs of cattle with respiratory disease or the nasopharynx of cattle without signs of respiratory disease, and the association of genotype 2 *M. haemolytica* with the lungs of cattle with respiratory disease. Section (**b**) shows a 2-by-2 table of the same 190 isolates categorized by their genotypes and the presence or absence of the conserved sequence backbone of ICE*Pmu1* and ICE*Mh1*. The table also shows the association of genotype 2 *M. haemolytica* with the conserved ICE backbone. Section (**c**) shows traits of genotype 1 and 2 *M. haemolytica*. The tree is a duplication of Fig. 1

Of the 155 isolates comprising group 1 of lung clinical isolates, 150 had genotype 2 and five had genotype 1. In comparison, of the 35 isolates comprising the nasopharyngeal non-clinical isolate group, six had genotype 2 and 29 had genotype 1 (Fischer’s exact test *p* <0.001, Odds Ratio = 145, CI_95%_ 36–647), (Fig. 4, Additional file 1). Thus, genotype 2 *M. haemolytica* strongly associated with the lungs of cattle with respiratory disease. Notably, a similar frequency of genotype 2 was also found in group 2 of lung clinical isolates. Of 162 isolates within the group, 152 had genotype 2 and ten had genotype 1.

### Association of genotype 2 *M. haemolytica* with ICE sequence

Of 190 isolates comprising group 1 of lung clinical isolates and the nasopharyngeal non-clinical isolate group, 154 of 156 genotype 2 isolates had sequence homologous to the conserved backbone of ICE*Pmu1*, ICE*Mh1*, and other related homologs. Conversely, 33 of 34 genotype 1 isolates did not (Additional file 1, Fig. 4, Fischer’s exact test *p* <0.001, Odds Ratio = 2541, CI_95%_ 186–91,615). Consequently, genotype 2 *M. haemolytica* associated with the conserved ICE backbone. A similar distribution was also observed in group 2 lung clinical isolates, where 150 of 152 genotype 2 isolates had the conserved ICE backbone, and eight of ten genotype 1 isolates did not.

### Association of genotype 2, subtype b (2b) *M. haemolytica* with antibiotic resistance genes encoded by ICE*Pmu1* or ICE*Mh1*

Nasopharyngeal non-clinical isolates were not included for association tests in this category as only six of them were genotype 2. Within 147 group 1 lung clinical isolates that were genotype 2 and subtyped with maximum likelihood significance, 80 were 2b and 67 were either 2c, 2d, or 2e. All 80 2b isolates were positive for one or more of the antibiotic resistance genes encoded by ICE*Pmu1* or ICE*Mh1*. Conversely, 66 out of the 67 genotype 2c, 2d, or 2e isolates were negative for all of the same antibiotic genes (Additional file 1, Fig. 5, Fischer’s exact test *p* <0.001, Odds Ratio = ∞, CI_95%_ 408–∞). Within the 147 group 2 lung clinical isolates that were genotype 2 and subtyped with maximum likelihood significance, 69 were 2b and 78 were either 2c, 2d, or 2e. All 69 2b isolates were positive for at least one of the antibiotic resistance genes encoded by ICE*Pmu1* or ICE*Mh1*. Conversely, all 78 isolates that were either 2c, 2d, or 2e were negative for the genes. Thus, 99% of the 194 tested isolates within the two lung clinical isolates groups that had antibiotic resistance genes encoded by ICE*Pmu1* or ICE*Mh1* were 2b, and the frequency of 2b isolates with one or more antibiotic resistance gene within both groups was 100%.

**Fig. 5:**
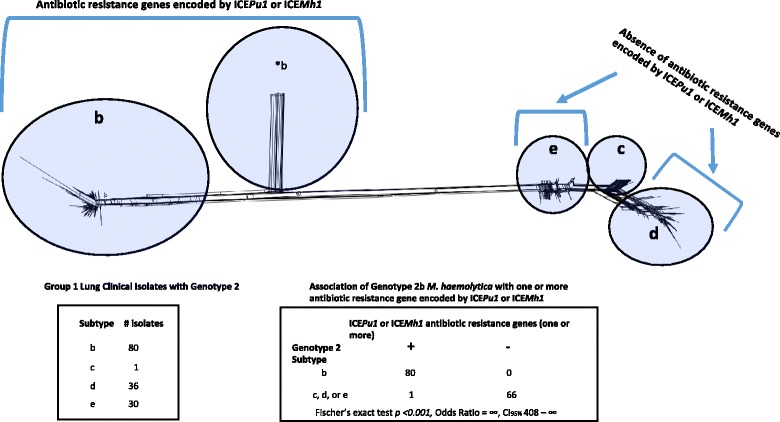
Association of *M. haemolytica* 2b with one or more antibiotic resistance gene found within the variable regions of ICE*Pmu1* or ICE*Mh1.* Group 1 lung clinical isolates that were genotype 2 (*N* = 147) were used for the association test. The single asterisk denotes genotype 2b that contains recombinant sequence. The network is a duplication of Fig. 3

### Linkage of antibiotic resistance genes in 2b isolates

Every 2b isolate in this study (*n* = 312) had the gene *tet(H)* (Additional file 1, Fig. 6), which encodes resistance to tetracycline [33], as well as a copy of its repressor gene *tet(R*), (data not shown). Additionally, there was complete linkage of the genes *aphA1, strB*, *strA* and *sul2* within all 2b isolates of the study. A total of 151 2b isolates were positive for *aphA1, strB*, *strA* and *sul2,* including 128 from groups 1 and 2 of lung clinical isolates. Conversely, 161 2b isolates were negative for *aphA1, strB*, *strA* and *sul2* (Additional file 1)*.* Collectively, these genes encode resistance to the aminogylcosides (kanamycin and neomycin (*aphaA1*) and streptomycin (*strA* and *strB*)), and the sulphonamide sulfamethoxazole (*sul2*), [23]. Within the lung clinical isolate groups, linkage was also observed between the genes *aadB*, *aadA15*, *floR*, *bla*_*OXA-2*_, *erm(42), msrE*, and *mphE*, although it was not perfect among the 37 2b isolates from both groups that were positive for some or all of the genes (Additional file 1)*.* Collectively, the genes encode for resistance to the aminoglycosides gentamicin (*aadB*), streptomycin and spectinomycin (*aadA15*), chloramphenicol and florfenicol (*floR*), some ß-lactams (*bla*_*OXA-*2_), and macrolides *erm(42), msrE*, and *mphE*).

**Fig. 6:**
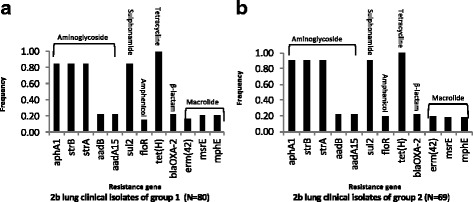
Frequencies of antibiotic resistance genes that mapped to variable regions of ICE*Pmu1* or ICE*Mh1* in *M. haemolytica* 2b. The frequencies represent: **a** group 1 lung clinical isolates and **b** group 2 lung clinical isolates

**Fig. 7:**
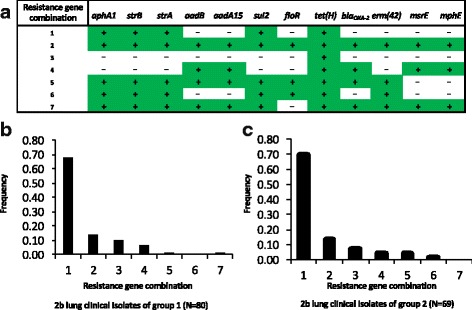
Unique combinations of antibiotic resistance genes and their frequencies in *M. haemolytica* 2b isolates from groups 1 and 2 of lung clinical isolates. Section (**a**) shows the seven combinations identified in this study. Plus signs indicate the gene is present, minus signs indicate the gene is not. Sections (**b**) and (**c**) show the combination frequencies in 2b isolates of groups 1 and 2 of lung clinical isolates, respectively

All 12 antibiotic resistance genes encoded by ICE*Pmu1* or ICE*Mh1* occurred as one of seven different antibiotic resistance gene combinations identified within all 2b isolates of this study (Fig. 7, Additional file 1). The *tet(H)* gene was present on all seven different combinations which accounted for 100% frequency of the gene in 2b isolates. While the genes *aphA1, strB*, *strA* and *sul2* were perfectly linked to each other, they co-occurred in five different combinations, with combination one being the most frequent within groups 1 and 2 of lung clinical isolates. Combination one consisted of *aphA1, strB*, *strA*, *sul2,* and *tet(H)* and was observed in lung clinical isolates which were obtained between 2002 and 2011 from diagnostic labs in three Canadian provinces and 27 US States. Combination one was additionally observed in 2b isolates of other groups that were obtained from cattle in Tennessee, Missouri, and Kentucky in 2013 (Additional file 1). Combination two, which had the second highest frequency in lung clinical isolate groups 1 and 2, contained all 12 antibiotic resistance gene (Fig. 7). This combination was found in 21 isolates that originated from diagnostic labs in 12 US states between the years 2003 and 2011. The earliest observation of this combination was in a 2003 isolate from a diagnostic lab in Oklahoma (Additional file 1). Thus, combination two, which encodes resistance to aminoglycosides, sulphonamide, phenicols, tetracycline, β -lactams, and macrolides, has been in circulation in the U.S. for over a decade.

## Discussion

A novel SNP-based classification system for *M. haemolytica* of North American cattle was developed in this study. Two major genotypes that both further divide into multiple subtypes were identified. The two genotypes significantly differ by their associations with the lungs of cattle with respiratory disease and ICE sequence homologous to the conserved backbone of ICE*Pmu1* and ICE*Mh1.* Additionally, just one subtype of genotype 2 associated with antibiotic resistance genes carried on ICE*Pmu1* or ICE*Mh1*. However, it is important to recognize how the isolates used to identify the associations differed from each other, that their history was not fully known regarding antibiotic exposure or lack thereof, and thus the context and some caveats regarding the associations identified in this study.

Both anatomical location and animal disease status differed between isolates of the two groups used for association tests involving genotypes 1 and 2, which were group 1 of lung clinical isolates and the group of non-clinical nasopharyngeal isolates. Group 1 of lung clinical isolates consisted of isolates from the lungs of cattle with respiratory disease, whereas the group of nasopharyngeal non-clinical isolates consisted of non-clinical isolates from the nasopharynx of cattle without signs of respiratory disease. Consequently, the genotype 2 association is with the lungs of cattle with respiratory disease, as opposed, for example, to just respiratory disease exclusively, or the lungs exclusively. By extension, the ICE sequence association with genotype 2 is also linked to the lungs of cattle with respiratory disease.

The two groups also differed by their depth of isolates across time and space. Group 1 of lung clinical isolates consisted of 155 isolates that represented 35 U.S. States and 5 Canadian Provinces from the years 2002–2011. Conversely, the group of nasopharyngeal non-clinical isolates represented three U.S. states and the year 2013. Given that genotype 2 was the predominant genotype of the lung clinical isolates, whereas genotype 1 was the predominant genotype of the nasopharyngeal non-clinical isolates, genotype 2 was well represented in the study. However, it is quite possible that genotype 1 North American *M. haemolytica* have more subtypes than those presently characterized, due to limited geographical and temporal representation of genotype 1 diversity in this study.

Additionally, for both groups used for association testing involving genotypes 1 and 2, the antibiotic treatment history of cattle from which the isolates originated was unknown. Given that the 155 cattle from which the group 1 lung clinical isolates were obtained were afflicted with respiratory disease at the time they were sampled, it is likely that some may have received antibiotic treatments prior to sampling*.* It is also not known whether any of the 35 cattle from which the nasopharyngeal non-clinical isolates were obtained received antibiotics prior to their sampling at sale barn locations. Disparate antibiotic exposure between isolates could have influenced ICE or genotype frequency differences between the two groups.

Although additional subtype diversity not observed in this study may exist within genotype 1 *M. haemolytica* of North America, it is unlikely that this diversity would associate with the lungs of diseased animals. This is because such diversity would have had notable frequency in either of the two groups of lung clinical isolates. The strength of the genotype 2 association with the lungs of cattle with respiratory disease was driven in large part by the classification of 150 of 155 (97%) group 1 lung clinical isolates as genotype 2. Between the two groups of lung clinical isolates, 302 of 317 (95%) of the isolates classified as genotype 2. Interestingly, of the 15 isolates that classified as genotype 1, six could not be subtyped with any statistical significance (Additional file 1). This may be because they gained alleles from genotype 2 that enhanced their pathogenicity through recombination, which may have complicated their placement within a particular subtype of genotype 1. To that end, recombination was detected within and across the two genotypes. However, it is also possible that the 15 isolates, while present in the lungs of cattle with respiratory disease, were not the causative agents of that disease. It is not uncommon to find *M. haemolytica* together with other bacteria in the lungs of cattle afflicted with respiratory disease [34, 35].

Although isolate exposure to antibiotics prior to entry in this study was unknown, 78 of 156 (50%) of the genotype 2 isolates used for ICE sequence association testing were negative for antibiotic resistance genes found in ICE*Pmu1* or ICE*Mh1*. Rather, they contained sequence homologous to the conserved backbone of ICE*Pmu1* and ICE*Mh1* (Additional file 1). Thus, antibiotic selection does not appear to account for 154 of 156 (99%) genotype 2 isolates having the conserved backbone ICE sequence. However, given that genotype 1 isolates were observed in this study with the backbone ICE sequence and aminoglycoside, sulfonamide, and tetracycline resistance genes, it is quite possible that the frequency of ICEs with antibiotic resistance genes could rise in genotype 1 *M. haemolytica* in the face of antibiotic exposure and selection.

While ICEs can have extensive host ranges, they are capable of preventing host cells from acquiring homologous ICEs by several different exclusion mechanisms [36]. Given that virtually all genotype 2 isolates had sequence homologous to the conserved backbone of ICE*Pmu1* and ICE*Mh1*, and that virtually all antibiotic resistance genes that mapped to variable regions of ICE*Pmu1* or ICE*Mh1* placed on the 2b background, ICEs with the conserved backbone of ICE*Pmu1* and ICE*Mh1* that are devoid of antibiotic resistance genes may be providing resistance to the acquisition of other ICE homologs with antibiotic resistance genes in 2e and 2d *M. haemolytica*. The same may also be true for 2c *M. haemolytica*. There were 93 isolates in this study that were 2c, and none of them had detectable antibiotic resistance genes. However, only two of those isolates belonged to lung clinical isolate groups and were epidemiologically unrelated, whereas the other 91 isolates were epidemiologically close to each other.

In stark contrast to all other *M. haemolytica* subtypes, every *M. haemolytica* 2b isolate in this study was positive for one or more antibiotic resistance gene in addition to the conserved backbone of ICE*Pmu1* and ICE*Mh1*. This may be because the ancestor of extant 2b *M. haemolytica* either did not originally have an ICE homologous to the conserved backbone of ICE*Pmu1* and ICE*Mh1* that lacked antibiotic resistance genes, or replaced it with a homolog that did. It is interesting that *tetH,* which encodes an efflux protein that provides tetracycline resistance [33], was identified on all seven combinations of resistance genes (Figs. 6 and 7). This infers it may be ancestral in its acquisition order compared to the other resistance genes. Oxytetracycline was licensed for use in the U.S. in product form in 1980 [16]. Additionally, prior to 1980, tetracycline compounds were used as growth promotants in cattle feed [37]. Therefore, selective pressure for tetracycline resistance may have been the result of cattle exposure to tetracyclines in the U.S. at therapeutic or sub therapeutic levels for decades.

Given that 2b *M. haemolytica* have seven antibiotic resistance gene combinations with the conserved ICE backbone, including one that encodes resistance to one or more antibiotic within six different classes (aminoglycosides, sulphonamides, amphenicols, tetracyclines, β –lactams and macrolides), and has been circulating in the U.S. since at least 2003, there has been considerable selection for this particular subtype to acquire antibiotic resistance. While all the subtypes of genotype 2 associate with the lungs of cattle with respiratory disease, it not known if one particular subtype is more virulent then another. If the ancestor of 2b *M. haemolytica* did have an ICE homolog to ICE*Pmu1* and ICE*Mh1* that lacked antibiotic resistance genes, enhanced virulence in comparison to other genotype 2 subtypes may have resulted in an increased exposure to antibiotic treatments, and selection pressures that overcame any preventative effects of the ancestral ICE against the acquisition of homologous ICEs with antibiotic resistance genes.

The clades that define genotypes 1 and 2 are separated by 13,941 tagging polymorphisms that are distributed throughout the *M. haemolytica* genome. The alleles of these polymorphisms are completely linked to each other in the genomes of the 1133 isolates included in this study, and define core genome backbones of genotypes 1 and 2 *M. haemolytica* (Additional files 2 and 3). While these tagging polymorphisms make it quite tractable to distinguish between the two genotypes, as any one of them could be used to classify an *M. haemolytica* as belonging to either genotype 1 or 2 (excluding for recombination events), they also complicate the identification of alleles that are biologically causative for the association of genotype 2 *M. haemolytica* with the lungs of cattle with respiratory disease. This is because alleles that biologically cause genotype 2 *M. haemolytica* to have that association are in linkage with thousands of other polymorphism alleles that tag the same genotype backbone.

Of the five subtypes defined in genotype 1, three of them (1b, 1e, 1i) showed signs of recombination through blocks of physically close tagging polymorphism alleles. Conversely, 1c and 1f had relatively few tagging polymorphisms that did not fall into blocks (Additional file 6). The lack of recombination in 1c and 1f indicates that they may be ancestral to 1b, 1e, and 1i, and that diversification in genotype 1 was driven by recombination on either the 1c or 1f background. Similarly, of the four subtypes defined in genotype 2, two of them had blocks of tagging polymorphisms that were indicative of recombination (2b and 2d), whereas two did not (2c and 2e), (Additional files 7 and 8). Thus, 2c and 2e appear ancestral to 2b and 2d, with recombination having driven diversification on either of their backgrounds to yield 2b and 2d. Consequently, *M. haemolytica* diversity in North America appears to be defined by a split of two major lineages with linear descent that was later followed by recombination and further diversification within both lineages, giving rise to the two major genotypes and their subtypes.

## Conclusions

North American *M. haemolytica* of cattle place into two major genotypes that are each defined by multiple subtypes. Genotype 2 *M. haemolytica* associate with the lungs of cattle with respiratory disease, and also with the conserved sequence backbone of ICE*Pmu1,* ICE*Mh1*, and other ICE homologs that does not contain antibiotic resistance genes. Genotype 2, subtype b *M. haemolytica* associate with antibiotic resistance genes that map to variable regions of ICE*Pmu1* and ICE*Mh1*. This particular subtype has been circulating in the U.S. since at least 2002 and may be more virulent to cattle than other genotype 2 subtypes. Tagging polymorphisms were identified that distinguish the two genotypes and their subtypes. Rapid identification of virulent antibiotic resistant strains of *M. haemolytica* in the field could facilitate interventions that prevent or minimize BRD outbreaks and ineffective antibiotic treatments, and consequently reduce therapeutic needs for treating BRD. Additionally, effective vaccines developed against genotype 2 *M. haemolytica* could further reduce the prevalence of BRD and subsequent needs for antibiotic treatments.

## Methods

### Isolates

The genomes of 1133 *M. haemolytica* isolates were sequenced and/or analyzed in this study. The isolates were categorized into two collections; Zoetis and KSU-USMARC, and four groups; lung clinical isolates group 1, lung clinical isolates group 2, nasopharyngeal non-clinical isolates, and clinical and non-clinical isolates (Table 1, Additional file 1). Groups 1 and 2 of lung clinical isolates consisted of 317 isolates that were part of a 10-year study of antimicrobial susceptibility of bacteria that cause BRD in the United States and Canada [14], (Additional file 1). These isolates were originally sent to Pfizer Animal Health (now Zoetis) by participating veterinary diagnostic laboratories, and their genomes were sequenced for this study with the permission of both Zoetis and the diagnostic laboratories that contributed the isolates. Group 1 of lung clinical isolates consisted of 155 isolates that originated from 35 U.S. States and 5 Canadian Provinces within the years of 2002–2011 (Additional file 1). Group 2 of lung clinical isolates consisted of 162 isolates that originated from 29 U.S. States and 3 Canadian Provinces. In order for an isolate to be included in a lung clinical isolate group, it had to originate from a different state, or a different year within a state from all other isolates in the group to minimize epidemiological relatedness.

Of the other 813 isolates used in the study, 810 were part of a longitudinal study comprised of 180 cattle that were first sampled for nasopharyngeal bacteria at the time of their purchase from sale barns located in Athens, Tennessee (*n* = 60 cattle), Richmond, Kentucky (*n* = 60 cattle), or Maryville, Missouri (*N* = 60 cattle) in the fall of 2013. The cattle were transported from the sale barns to a research feeding facility in Kansas where half received a metaphylactic treatment for the control of BRD (gamithromycin (6 mg/kg, subcutaneously administered), and half received a saline control. The animals were then monitored for BRD and periodically sampled over 28 days for *M. haemolytica* and other bacteria from nasopharyngeal swabs and/or bronchoalveolar lavage fluid and monitored for signs of BRD as previously described [34], (Additional file 1).

The 35 isolates comprising the group of nasopharyngeal non-clinical isolates from the KSU-USMARC collection were selected for minimal epidemiological relatedness to each other based on geographical origin (Table 1, Additional file 1). Each of the 35 isolates originated from the nasopharynx of a different animal that did not have clinical signs of BRD. Ten of the cattle sampled were at a sale barn in Athens, Tennessee, 12 were at a sale barn in Maryville, Missouri, and 13 were at a sale barn in Richmond, Kentucky. The isolates were obtained from the cattle prior to their transport to the Kansas research feeding facility and their genomes were sequenced for this study.

The group of clinical and non-clinical isolates consisted of 781 isolates of which many were epidemiologically close, or directly related to each other. The genomes of 505 isolates from this group were sequenced for this study. The genomes of the remaining 276 isolates were sequenced as previously described [29, 30], (Additional file 1).

### Isolate culture from field collected samples and *M. haemolytica* identification

Isolates from the Zoetis collection were originally identified as *M. haemolytica* by diagnostic labs located in the U.S. or Canada, and were further characterized or confirmed as *M. haemolytica* as necessary [14]. Additionally, the isolates were re-confirmed as *M. haemolytica* in 2013 via the OmniLog microbial identification system using GEN III chemistry as per the manufacturer’s protocol (Biolog, Inc., Hayward, CA, USA).

Nasopharyngeal non-clinical isolates were cultured from two deep nasopharyngeal swabs that were collectively taken from the left and right nasopharynx of each animal with double guarded sterile uterine swabs (VetOne, Boise, Idaho, USA). Swabs from each nasopharynx of an animal were either combined in sample tubes containing liquid Amies media (Becton, Dickinson and Company, Franklin Lakes, New Jersey, USA) and transported to the US Meat Animal Research Center at 4 °C, or combined in liquid Amies media with 20% glycerol, frozen at −80 °C, and subsequently transported to the US Meat Animal Research Center. Aliquots of the media were spread plated onto both Chocolate agar and Brain Heart Infusion (BHI) agar plates containing 5% sheep blood (Hardy Diagnostics, Santa Maria, CA, USA) and cultured 16–20 h at 37 °C with 5% CO_2_. Hemolysis, or lack thereof, on BHI blood agar plates was not an identification criterion for *M. haemolytica,* as not all strains show *β*-hemolysis on culture plates containing bovine or ovine blood [38, 39]. Colonies that resembled *M. haemolytica* by morphology were cultured for 16–20 h in deep 96-well blocks containing 1 ml of liquid BHI (Becton, Dickinson and Company, Franklin Lakes, NJ, USA) per well at 37 °C without CO_2_ and subsequently frozen at −80 °C with 10% glycerol. The isolates were later cultured twice on chocolate agar plates and colonies from the second culture were identified as *M. haemolytica* via Gen III as described above.

Regarding isolates of the clinical and non-clinical group, those obtained from animals not showing clinical signs of BRD were obtained and identified as *M. haemolytica* as described for the group of nasopharyngeal non-clinical isolates. Isolates within the clinical and non-clinical group that came from animals diagnosed with respiratory disease were either obtained from deep nasopharyngeal swabs or bronchoalveolar lavage samples as previously described [34]. The clinical isolates were cultured on trypticase soy agar plates with 5% blood, chocolate agar plates, and MacConkey agar plates in 5% CO_2_ for 18–24 h as previously described [34]. The clinical isolates were identified as *M. haemolytica* via matrix assisted laser desorption ionization time-of-flight mass spectrometry (MALDI-TOF MS), per the manufacturer’s protocol (Bruker Daltonics, Billerica, MA, USA).

### DNA purification, library construction, and sequencing

The genomes of 857 *M. haemolytica* isolates were sequenced for this study. This included all 317 isolates in the Zoetis collection, all 35 isolates in the group of nasopharyngeal non-clinical isolates, and 505 of the 781 isolates in the group of clinical and non-clinical isolates. Genomic sequencing of the remaining 276 isolates in the group of clinical and non-clinical isolates has been described elsewhere [29, 30]. The sequence data of those 276 isolates were included in this study for nucleotide polymorphism identification and downstream analyses.

For DNA purification, isolates were cultured in deep 96 well blocks containing 1 mL BHI per well at 37 °C without supplemental CO_2_ for 16–20 h. DNA was purified from the cultures using MO BIO UltraClean® -htp 96 Well Microbial DNA kits per the manufacturer’s instructions (Carlsbad, CA, USA). Resulting DNA samples were quantified with a Promega Quantus Fluorometer and QuantiFluor Dyes® per the manufacturer instructions (Promega, Madison, WI, USA). Illumina-based Nextera XT DNA Library kits were prepared in batches of 96 as per the manufacturer’s instructions, with Nextera XT Index Kits (V1) set A which provided 96 unique index combinations (Illumina, San Diego, CA, USA). Given that the Nextera XT DNA Library kit protocol involved a PCR step, pre- and post-PCR procedures were conducted in physically separated labs to avoid aerosol contamination of samples, reagents, and libraries. Subsequent to their construction, batches of 96 libraries were combined, and paired-end sequencing was conducted on an Illumina MiSeq instrument with either MiSeq Reagent Kit version 2 (2 × 250 bp, 500 cycles) or version 3 (2 × 300 bp, 600 cycles), (Illumina, San Diego, CA, USA).

### Quality control checks of isolate sequence

Sequence coverage for each isolate was determined to ensure that a minimum of 10× sequence per genome was attained. The coverage was calculated using total bases of sequence per isolate divided by 2.6 million, which is the typical genome size for known *M. haemolytica* isolates in GenBank. Any isolate that did not have 10× sequence per genome was re-sequenced to achieve that coverage or higher.

The DNA sequence of each isolate was also checked for evidence of contamination and/or isolate misidentification using Geneious software and a series of BLAST analyses. Geneious (version 8.1.5), [40], was used to map the sequence reads from each isolate using the Geneious mapper program with Medium/Fast Sensitivity and fine tuning of up to 5× iterations. The reads were mapped to an available complete, finished, circular reference genome of *M. haemolytica* strain USDA-ARS-USMARC-183 which was isolated from a 1991 BRD case in Kansas, USA ([28], GenBank: CP004752). Because the Illumina platform generated paired-end reads of clonal sequence [41], the mapped reads could be checked for the presence of nucleotide polymorphisms where both alleles were observed between reads from the same isolate, and were not the result of mapping or sequencing errors. Detection of these polymorphisms was an indication that more than one type of *M. haemolytica*, or bacterial species was represented in the library. Any *M. haemolytica* isolate found to be represented by a library that yielded sequences indicative of different strain types of *M. haemolytica*, or other bacterial species, was re-cultured for isolation of all discernable *M. haemolytica* types. The resulting isolates were subsequently sequenced. Additionally, for each isolate sequenced in this study, full length 16S rDNA and the leukotoxin gene (*lktA*), as well as random regions selected throughout the genome were compared to *M. haemolytica* reference genomes present in NCBI GenBank using BLASTN [42], for *in silico* confirmation that the isolates were *M. haemolytica.*

### Genome-wide nucleotide polymorphism identification and genotyping

MiSeq-produced sequence reads of the 857 *M. haemolytica* isolates sequenced in this study, as well as those of the 276 previously sequenced *M. haemolytica,* were mapped to the genome of *M. haemolytica* strain USDA-ARS-USMARC-183 using Bowtie 2 (v2,2,1) with default and the “very-sensitive-local” arguments at Intrepid Bioinformatics Inc (Louisville, KY, USA). Standard alignment post-processing of the mapped reads which included the marking of PCR duplicates and realignment of insertion deletion polymorphisms was done via done Genome Analysis Toolkit (GATK) v1.5-32-g2761da9. “Singleton” nucleotide polymorphisms, where the minor allele was observed in only one isolate, as well as insertion deletion polymorphisms, were not included in the dataset. All isolates were initially genotyped for nucleotide polymorphisms identified throughout the genome. Nucleotide polymorphisms identified within integrated phage sites and duplicated genomic regions of strain USDA-ARS-USMARC-183 were not included in subsequent analyses due the inherent difficulty in accurately mapping short sequence reads to repetitive sequence that can create polymorphism artifacts [43, 44].

### Identification of ICE sequence

ICE sequences with or without the macrolide resistance genes *erm*(42), *msr*(E), and *mph*(E) have been reported for the 276 *M. haemolytica* isolates included in this study that were previously sequenced [29]. The 857 *M. haemolytica* isolates sequenced in this study, and the additional 276 previously sequenced isolates, were queried for ICE and antibiotic resistance gene sequences by mapping their sequence reads to three homologous ICE sequences using Geneious (version 8.1.5). The three ICEs were ICE*Pmu1,* which was identified within the genome of *P. multocida* 36950 (GenBank: CP003022), ICE*Mh1*, which was identified within the genome of *M. haemolytica* isolate M42548 (GenBank: CP005383), and a putative ICE identified in *M. haemolytica* isolate USDA-ARS-USMARC-183 that is homologous to the conserved sequence backbone of ICE*Pmu1* and ICE*Mh1* and does not contain antibiotic resistance genes [23]. The presence or absence of ICE sequence, as well as antibiotic resistance genes present in ICE*Pmu1* and ICE*Mh1* was determined for each isolate based on the observation of reads mapped to annotated regions of those reference sequences.

### Trees and networks constructed from concatenated nucleotide polymorphism genotypes

For each isolate, concatenated nucleotide polymorphism genotypes were created from their corresponding alleles of genome-wide polymorphisms identified in this study. A Neighbor-Joining tree was generated from concatenated nucleotide polymorphism genotypes of all 1133 isolates involved in this study (Fig. 1). The nucleotide polymorphisms used to generate the concatenated nucleotide polymorphism genotypes for generation of the tree each had allele genotypes scored for 95% or more of the isolates. The alleles were formatted into an alignment in MacVector (version 14.5.1), [45] using clustal W. A Neighbor-Joining tree was generated from the alignment using the following programs: Seqboot, Dnadist, Neighbor, and Consense which are all part of PHYLIP (version 3.69), [46]. An F84 model of substitution with a transition transversion ratio of 2 was used to generate the tree, which was bootstrapped 100 times. The tree was viewed using the program Dendroscope (version 3.5.7), [47, 48].

A Neighbor-Joining network was generated from the concatenated nucleotide polymorphism genotypes of 521 genotype 1 isolates (Fig. 2), and another from those of 600 genotype 2 isolates (Fig. 3). The nucleotide polymorphisms used to generate the networks had allele genotypes scored for 90% or more of the isolates. The nucleotide polymorphism genotypes of the 521 genotype 1 isolates represented in Fig. 2 were tested for recombination using a phi test [49], in SplitsTree4 (version 4.12.3), [50], as were those of the 600 genotype 2 isolates represented in Fig. 3. The networks were also made in SplitsTree4 with an F84 model of substitution and a transition/transversion ratio of 2. Support for clusters identified in the networks was determined using IQ-TREE (version 1.3.10), which is a maximum-likelihood phylogeny platform [51]. For each isolate group, ModelFinder was used within IQ-TREE to find the optimal model of substitution. For the networks of Figs. 2 and 3, GTR + G and SYM + G models of substitution were used, respectively, to generate the maximum-likelihood trees. Support within the trees was determined in IQ-TREE with 1000 dataset replicates using SH-aLRTs, [52], and ultrafast bootstraps [53].

### Statistical testing

Isolates comprising the two following groups were used for statistical testing; lung clinical isolates group 1 (*n* = 155), and the group of nasopharyngeal non-clinical isolates (*n* = 35). Two-way contingency table analyses were conducted to test for an association between genotype 2 *M. haemolytica* with 1) the lungs of diseased animals, and 2) the presence of ICE sequence homologous to the conserved sequence backbones of ICE*Pmu1,* ICE*Mh1* and other homologs (http://statpages.info/ctab2x2.html), [54]. Additionally, a 2-way contingency table analysis was conducted to test for an association between genotype 2b *M. haemolytica* and antibiotic resistance genes found in variable regions of ICE*Pmu1* or ICE*Mh1*. Only group 1 lung clinical isolates that were genotype 2 with subtypes supported by SH-aLRT and ultrafast bootstrap values were used for this analyses (*n* = 147). The group of nasopharygeal non-clinical isolates was not included due to the low frequency of genotype 2 *M. haemolytica* in the group (*n* = 6).

## Additional files

Additional file 1: Table of *M. haemolytica* isolates used in this study. For each isolate, the table contains information regarding a Minilims identifier number for the isolate and for the isolate library, state or province of origin, year of isolation, the collection the isolate belongs to, an AHDRCC identifier number, the group the isolate belongs to, use in the study, genotype and subtype designation, ICE and antibiotic resistance gene sequence information. (XLSX 148 kb)
Additional file 2: Table of nucleotide polymorphism allele locations and genotypes used to initially type all 1133 *M. haemolytica* isolates (first table). Due to the size of the dataset this is the first of two tables that contain the genotypes (Additional files 2 and 3). The table contains the locations and allele scores for 11,306 of 16,447 nucleotide polymorphisms that were used to generate the tree of Fig. 1. Each polymorphism was unambiguously scored in 95% or more of the isolates. (ZIP 4344 kb)
Additional file 3: Table of nucleotide polymorphism allele locations and genotypes used to initially type all 1133 *M. haemolytica* isolates (second table). Due to the size of the dataset this is the second of two tables that contain the genotypes (Additional files 2 and 3). The table contains the locations and allele scores for 5141 of 16,447 nucleotide polymorphisms that were used to generate the tree of Fig. 1. Each polymorphism was unambiguously scored in 95% or more of the isolates. (CSV 11580 kb)
Additional file 4: Table of nucleotide polymorphism allele locations and genotypes used to type genotype 1 *M. haemolytica* isolates (first table). Due to the size of the dataset, this is the first of two tables that contain the genotypes (Additional files 4 and 5). The table contains the locations and allele scores for 11,712 of the 16,888 nucleotide polymorphisms used to type genotype 1 *M. haemolytica* and construct the network of Fig. 2. Each polymorphism was unambiguously scored in 90% or more of the isolates. (CSV 12221 kb)
Additional file 5: Table of nucleotide polymorphism allele locations and genotypes used to type genotype 1 *M. haemolytica* isolates (second table). Due to the size of the dataset, this is the second of two tables that contain the genotypes (Additional files 4 and 5). The table contains the locations and allele scores for 5176 of the 16,888 nucleotide polymorphisms used to type genotype 1 *M. haemolytica* and construct the network of Fig. 2. Each polymorphism was unambiguously scored in 90% or more of the isolates. (CSV 5410 kb)
Additional file 6: Table of nucleotide polymorphisms with both alleles observed in genotype 1 *M. haemolytica* isolates. The table shows informative and tagging polymorphisms for the subtypes of genotype 1. (XLSX 87 kb)
Additional file 7: Table of nucleotide polymorphism allele locations and genotypes used to score genotype 2 *M. haemolytica* isolates (first table). Due to the size of the dataset, this is the first of two tables that contain the genotypes (Additional files 7 and 8). The table contains the locations and allele scores for 16,246 of 24,475 nucleotide polymorphisms used to type genotype 2 *M. haemolytica* and construct the network of Fig. 3. Each polymorphism was unambiguously scored in 90% or more of the isolates. (ZIP 3413 kb)
Additional file 8: Table of nucleotide polymorphism allele locations and genotypes used to score genotype 2 *M. haemolytica* isolates (second table). Due to the size of the dataset, this is the second of two tables that contain the genotypes (Additional files 8 and 9). The table contains the locations and allele scores for 8229 of 24,475 nucleotide polymorphisms used to type genotype 2 *M. haemolytica* and construct the network of Fig. 3. Each polymorphism was unambiguously scored in 90% or more of the isolates. (CSV 9995 kb)
Additional file 9: Table of nucleotide polymorphisms with both alleles observed in genotype 2 *M. haemolytica* isolates. The table shows informative and tagging polymorphisms for the subtypes of genotype 2. (XLSX 131 kb)

## Abbreviations

BRD: Bovine respiratory disease
ICE: Integrative and conjugative element
ICE*Mh1*: Integrative and conjugative element identified in *M. haemolytica*
ICE*Pmu1*: Integrative and conjugative element identified in *P. multocida*
MALDI-TOF MS: Matrix assisted laser desorption ionization time-of-flight mass spectrometry
PFGE: Pulsed-field gel electrophoresis
SH-aLRT: Shimodaira-Hasegawa approximate likelihood ratio test
